# Promoter Screening from *Bacillus subtilis* in Various Conditions Hunting for Synthetic Biology and Industrial Applications

**DOI:** 10.1371/journal.pone.0158447

**Published:** 2016-07-05

**Authors:** Yafeng Song, Jonas M. Nikoloff, Gang Fu, Jingqi Chen, Qinggang Li, Nengzhong Xie, Ping Zheng, Jibin Sun, Dawei Zhang

**Affiliations:** 1 Tianjin Institute of Industrial Biotechnology, Chinese Academy of Sciences, Tianjin 300308, P. R. China; 2 Key Laboratory of Systems Microbial Biotechnology, Chinese Academy of Sciences, Tianjin 300308, P. R. China; 3 National Engineering Research Center for Non-food Biorefinery, State Key Laboratory of Non-food Biomass Energy and Enzyme Technology, Nanning 5300074, P. R. China; 4 Guangxi Biomass Industrialization Engineering Institute, Guangxi Academy of Sciences, Nanning 530007, P. R. China; Imperial College London, UNITED KINGDOM

## Abstract

The use of *Bacillus subtilis* in synthetic biology and metabolic engineering is highly desirable to take advantage of the unique metabolic pathways present in this organism. To do this, an evaluation of *B*. *subtilis*’ intrinsic biological parts is required to determine the best strategies to accurately regulate metabolic circuits and expression of target proteins. The strengths of promoter candidates were evaluated by measuring relative fluorescence units of a green fluorescent protein reporter, integrated into *B*. *subtilis’* chromosome. A total of 84 predicted promoter sequences located upstream of different classes of proteins including heat shock proteins, cell-envelope proteins, and proteins resistant against toxic metals (based on similarity) and other kinds of genes were tested. The expression levels measured ranged from 0.0023 to 4.53-fold of the activity of the well-characterized strong promoter P43. No significant shifts were observed when strains, carrying different promoter candidates, were cultured at high temperature or in media with ethanol, but some strains showed increased activity when cultured under high osmotic pressure. Randomly selected promoter candidates were tested and found to activate transcription of thermostable β-galactosidase (bgaB) at a similar level, implying the ability of these sequences to function as promoter elements in multiple genetic contexts. In addition, selected promoters elevated the final production of both cytoplasmic bgaB and secreted protein α-amylase to about fourfold and twofold, respectively. The generated data allows a deeper understanding of *B*. *subtilis’* metabolism and will facilitate future work to develop this organism for synthetic biology.

## Introduction

Metabolic engineering seeks to increase the synthesis of desired products *de novo* and by modification of existing metabolic pathways or by optimization of appropriate genetic elements [1–4]. The use of different biological elements may help to tune expression to achieve the desired production level, provided that cell metabolism remains co-ordinated [5–8]. Some organizations, *e*.*g*. the Genetically Engineered Machine (iGEM) Foundation, have tested the efficiency of sequence elements to function as interchangeable components or biobricks that can be used to build biological systems. The goal is to assemble libraries of these elements that can be applied for the engineering of living cells.

Current efforts in synthetic biology have focused on the evaluation of cis-sequences including promoters, ribosome binding sites (RBS), and terminators in both *Escherichia coli* and *Saccharomyces cerevisiae* [9–12]. There are several successful examples of changing promoters to alter expression in prokaryotic and eukaryotic cells. Hal Alper *et al* constructed a promoter library based on the bacteriophage P_L_-λ promoter that was generated using error-prone PCR. The library was tested for promoter strength by measuring the downstream expression of *gfp* and the chloramphenicol acetyltransferase gene, and those that exhibited a linear relationship between promoter strength and reporter were selected. They then expressed a series of promoter-*dxs* (deoxy-xylulose-P synthase) constructs in a recombinant *E*. *coli* strain overexpressing genes (*ispFD* and *idi*) of the isoprenoid synthesis pathway, and observed the linear response of lycopene yield to promoter strength. [13]. In a second example, a strong and tunable promoter library was obtained that showed a range of 400-fold at the mRNA level [14]. This library was created by combining various copies of upstream activation sequences with the native promoter AOX1 from *Yarrowia lipolytica*. The final expression of humanized Renilla GFP (hrGFP) in *Y*. *lipolytica* was increased eightfold over the activity of the original endogenous promoter [14]. In this system, both a high output of heterologous protein and a strictly controlled metabolic pathway were engineered [14]. In another report, a set of insulated promoters, differing in strength and context-independent behavior, was designed and applied for controlled protein production. The properties of those promoter devices in one test context were predictive of those properties in a new context allowing steady-state protein production regulated by transcriptional regulation [15]. 195 native or synthetic promoters and 192 RBSs were characterized for their ability to drive the expression of superfold GFP (sfGFP) in *Streptomyces venezuelae* [16]. Next, an insulator sequence, RiboJ, was introduced to reduce interference between promoters and RBSs and these combinations were again tested by examination of sfGFP levels. The insulator element RiboJ is a DNA sequence that contains both a ribozyme and hairpin acts to help expose the RBS [16]. These synthetic modular regulatory elements were then inserted upstream of the lycopene biosynthetic cluster in *S*. *avermitilis*. The correlation between lycopene production and promoter strength, elevated lycopene titer as well, confirmed the utilization and feasibility of these expression cassettes and paved the way for further application of these promoter elements in system biotechnology [17].

*B*. *subtilis* shows significant advantages as a host for protein expression due to its efficient secretion capacity and because it is a generally recognized safe organism. It is used for applications in the detergent, textile, and pharmaceutical industries [18–20]. However, due to unsuitable vectors containing weak promoters, RBS sequences, terminators, incompatible antibiotic genes, or insufficient plasmid copy numbers [21], many heterologous proteins are produced with only low yields using these vectors. In addition, overexpressing genes of secretory components is time-consuming and inefficient, particularly due to the complexity of the secretion mechanisms [22,23]. There are recent reports from large-scale omic-studies of this Gram-positive bacterium [24–26] that discussed the variation of proteins or transcripts after different conditions of cultivation. However, they compared only a few examples for specific promoter strength and did not perform a comprehensive analysis. Some promoters were discovered or modified by optimization of their key elements [27] for application in target expression systems, but no systematic promoter candidate strength evaluation has been performed using experimental assessment of reporter gene transcription. Some accessible omic-data about promoter candidates was from *B*. *thuringiensi*s rather from *B*. *subtilis*. In that study, Wang *et al* systematically identified 1203 active promoter candidates through analysis of genome-wide transcription start sites based on RNA-seq data. Additionally, they further evaluated the characteristics of 20 highly active promoters combined with the corresponding 5’-UTR to screen the highly active promoter-5’-UTR DNA region complex by directing the expression of reporter gene *lacZ* [28]. Therefore, an efficient and facile approach for achieving desired production goals is selection of a suitable promoter element to strictly control related protein expression levels and to allow precise and functional modularity [29].

Here, we constructed a promoter-probe vector with GFP as a detection target and measured expression levels. We wanted to identify stress-activated promoters that could direct high-level expression under specific stress conditions, like heat shock, high salt, or ethanol to stimulate membrane stress. More than 80 promoter candidates that showed high transcription levels under multiple conditions were selected and classified according to the encoded proteins located downstream from the promoters [30]. Promoter candidate activities, relative to that of the constitutive strong promoter of P43, [31], ranged from 0.0023 to 4.53, which spanned a ~1960-fold range. After heat shock, salt, or ethanol treatment, they showed changed RFU levels when compared with normal cultivation, however, the changes were less than expected. The strong promoter P*trnQ* exhibited higher activity than P43 in driving the transcription of both cytoplasmic and secretory proteins (about four- and two-fold, respectively), enabling normalized measurement of the promoter candidates’ strengths.

## Materials and Methods

### Strains and plasmids

*E*. *coli* DH5α was used as cloning host. The plasmid-free strain *B*. *subtilis* 1A751 was used as the main host for the promoter library. Tested promoter candidates were amplified from *B*. *subtilis* 168 genomic DNA. The function and relevant properties of strains and plasmids are listed in Table 1.

**Table 1:** Strains and plasmids used in this study.

Strains and plasmids	Relevant properties	Source
Strains		
*E*. *coli* DH5α	F- F80 *lacZ Δ M15 recA endA1Δ(lacZYA-argF) U169 deoR gyrA96 thi-I hsdR17 supE44 relAI*	Our laboratory
*B*. *subtilis* 1A751	*eglSΔ102bglT/bglSΔEV aprE nprE his*	Our laboratory
*B*. *subtilis* 168		Our laboratory
Plasmids		
ECE164	*gfpmut1*	BGSC
pDL	Promoterless, *bgaB*	BGSC
pDL-GFP	pDL-derived, *bagB* was substituted by *gfpmut1*, without promoter	This work
pDL-GFP-PC	pDL-derived, promoter candidates	This work
pMA5-P43	P43 promoter, α-amylase	Our laboratory
pMA5-P*trnQ*	P*trnQ* promoter, α-amylase	This work

BGSC, Bacillus Genetic Stock Center, USA

### Construction of the promoter-probe vector and vectors carrying different promoter candidates and pMA5 containing P*trnQ*

To simplify construction and utilize a strategy that did not rely on restriction enzymes, we used prolonged-overlap-PCR to replace the *bgaB* gene of pDL with *gfpmut1* [32]. Primers used in this procedure are listed in Table 2.

**Table 2:** Oligonucleotides used in this study.

oligonucleotides	sequences
gfp-f	acgaaaattagctagggggaataattatgagtaaaggagaagaacttttcac
gfp-r	ccaactgtcggaacgagacttctctatttgtatagttcatccatgccatgtg
pdlVF	cacatggcatggatgaactatacaaatagagaagtctcgttccgacagttgg
pdlVR	gtgaaaagttcttctcctttactcataattattccccctagctaattttcgt
promoter candidate X-s	Listed in S1 Table
promoter candidate X-a	Listed in S1 Table
ecoli-s	ggatttgagcgtagcgaaaaatcc
ecoli-a	cgggcatggcactcttgaaaaag
bacillus-s	ggagtgtcaagaatgtttgcaaaacg
bacillus-a	ctttttcaagagtgccatgccc
qPCR-gfp-up	ctgtcagtggagagggtgaaggtgatgc
qPCR-gfp-down	ccttcgggcatggcactcttgaaaaag
qPCR-gap-up	gcaaacggcgcaaggcagtttgttg
qPCR-gap-down	cttcacaaaacgcgcagacgctgc

Promoter candidate fragments were prepared by PCR amplification using *B*. *subtilis* 168 genomic DNA as template, PrimerSTAR Max DNA Polymerase mix containing dNTP and DNA polymerase (TaKaRa, Japan), and primers (Genwiz, China) listed in Table 2, according to the instructions of the manufacturer. 2000 bp upstream of the start codon of the target protein were amplified as the promoter candidates. The enzyme digestion sites in the vector 5’ and 3’ ends were BamHI and KpnI (BamHI/EcoRI, EcoRI/KpnI). After purification of the digested promoter candidates (E.Z.N.A.^™^ Cycle Pure Kit 200, Omega Bio-tek, Inc., USA) and the vector enzyme digestion products, the ligation was performed using T4 ligase (Thermo Fisher Scientific, USA) according to the instructions of the manufacturer and transformed into *E*. *coli* DH5α. Putative candidate colonies carrying cloned promoter fragments were identified by colony PCR before being validated by sequencing (Genwiz, China).

### Culture media and expression conditions

Cells were incubated aerobically at 37°C and 220 rpm, in LB media. Antibiotics were added where appropriate (ampicillin at 100 μg/mL, kanamycin at 40 μg/mL, chloramphenicol at 5 μg/mL). Cells for the GFP fluorescence intensity assays were incubated in LB in 96-well micro-plates.

### Whole cell fluorescence measurements

Expression of GFPmut1 was monitored using whole-cell fluorescence under the multimode microplate reader (SpectraMax M5) at an optical density of 0.4–0.6 for λ = 600 nm (OD_600_) for the different transformants. Cells were centrifuged at 4000 rpm for 10 minutes, the supernatant was discarded, and cells were resuspended in an equal volume of double distilled water. The extinction and emission wavelength were set at 488 nm and 523 nm, respectively. *B*. *subtilis* 1A751 without chromosomal *gfp* was defined as the negative control. Standard deviations are based on a minimum of three statistically independent experiments.

### Fluorescence-activated cell sorter (FACS) analysis

Fluorescence-activated cell sorter (FACS) can process tens of thousands of individual cells within a few seconds and obtain many parameters at the same time. In order to guarantee the accuracy of the detected results, we measured the RFU of some selected samples using FACS. For the FACS analysis, cells were grown for 6.5 h at 37°C and then were centrifuged at 12000 rpm for 2 minutes. After discarding the supernatant, cells were resuspended in phosphate-buffered-saline (PBS). Samples were analyzed using a FACS Aria Cell Sorting System (Beckman, MoFlo XDP, USA) with a 100 mW, 488 nm coherent sapphire solid state laser for GFP. *B*. *subtilis* 1A751 without chromosomal *gfp* was defined as the negative control.

### RNA isolation, reverse transcriptase PCR (RT-PCR), and real-time fluorescence quantitative PCR

Overnight cultures were inoculated at a ratio of 1:100 into 5 mL LB. After growing for 4 h and 45 min at 37°C, cells were collected for RNA isolation. For those cells containing promoter candidates from heat shock proteins, cultures were grown at 43°C instead of 37°C for 15 min before testing. Similarly, cells containing promoter candidates from cell-envelope proteins were tested by incubation in LB media for 4 h and 30 min followed by addition of ethanol to a final concentration of 4% (v/v). Cells containing promoter candidates from proteins that confer resistance to toxic metals were tested by incubation in LB media for 4 h and 30 min followed by addition of CoCl_2_ to a final concentration of 8 mM. 5 mL cultures were used for RNA extraction according to the manufacturer’s instructions (TIANGEN, China) and the PrimeScript RT reagent Kit (TaKaRa) was used to extract cDNA. Real-time fluorescence quantitative PCR (qRT-PCR) was performed with FastStart Universal SYBR Green Master (ROX) (Roche, Germany) using a 7500 system (Applied Biosystems, USA) following the manufacturer’s instructions. Based on the 2^-ΔΔCt^ method, we calculated the gene expression level by using housekeeping gene *gap* as reference gene. PCR conditions were the following: reverse transcription at 50°C for 2 min, then denaturation at 95°C for 10 min, followed by 40 cycles of denaturation at 95°C for 15 s and then annealing and extension at 60°C for 1 min. The primer sequences for *gap* and *gfp* amplification are listed in Table 2.

### Measurement of BgaB activity and α-amylase activity

The BgaB activity assay was carried out as previously described [33]. Samples were collected after incubating in LB media for 4 h and 45 min with an inoculation ratio of 1:100. The α-amylase activity assay was performed as described previously [13]. 1 mL supernatants of culture samples after centrifugation were collected to perform sodium dodecyl sulfate polyacrylamide gel electrophoresis (SDS-PAGE) (NuPAGE 10% Bis-Tris Gel, Commassie Brilliant Blue, Invitrogen Life Technologies, USA).

## Results

### Strength of different promoter candidates from *B*. *subtilis*

**Fig 1:**
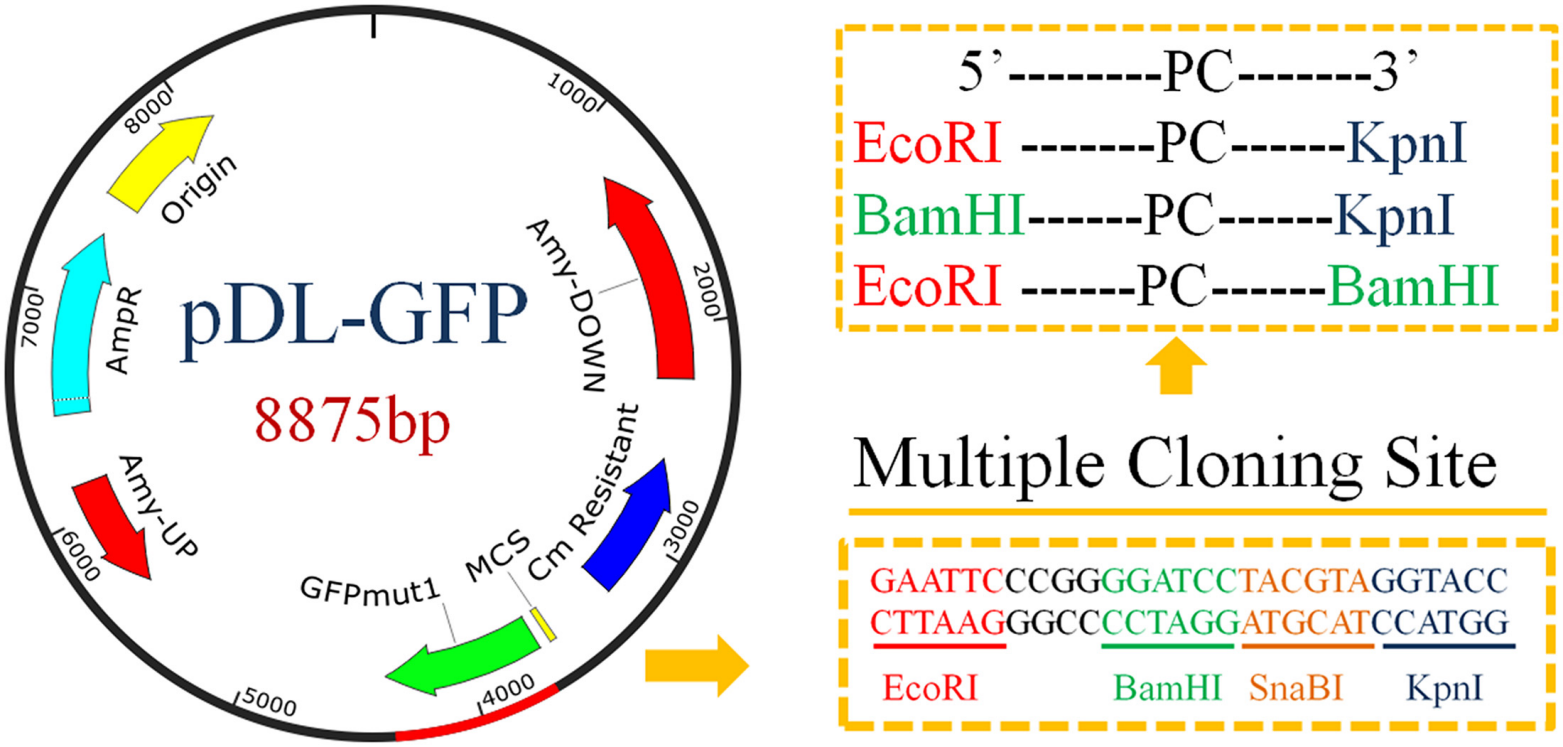
Scheme of the constructed promoter-probe vector pDL-GFP showing multiple cloning sites (MCS) and restriction enzyme recognition sites. The boxes show the 5’ and 3’-end enzymes used for ligation of promoter candidates (PC). The left side shows the backbone of the integrated plasmid pDL-GFP and the main genes. On the right side at the bottom the sequences and enzyme sites within it are shown.

To measure promoter candidates’ strengths precisely, it is ideal if single copies of the reporter gene can be inserted into the genome to avoid complications due to heterogeneous numbers of plasmids in each bacterium. An inaccurate distribution of plasmid copy numbers may even occur within the same population of cells, thus single copy gene replacements or insertions are preferred over plasmids [34]. pDL is a promoter-probe vector carrying sequences corresponding to the genomic sequences upstream and downstream of *amyE*, which can be used to facilitate double over-crossing with the chromosome [35]. Loss of *amyE* has no detectable effect on growth. However, the reporter gene in pDL is *bgaB*, which requires complex detection procedures. Thus, to simplify screening, we exchanged this gene with *gfp* [36]. The pDL plasmid carrying the cloned promoter fragment upstream of GFP cassette was linearized with PstI and integrated into the *B*. *subtilis*’ genome through homologous recombination. Prolonged overlap extension PCR approaches were used to generate the pDL-GFP vector (Fig 1) which obviated the need to look for compatible restriction enzyme sites and allowed high efficiency transformation [37].

**Fig 2:**
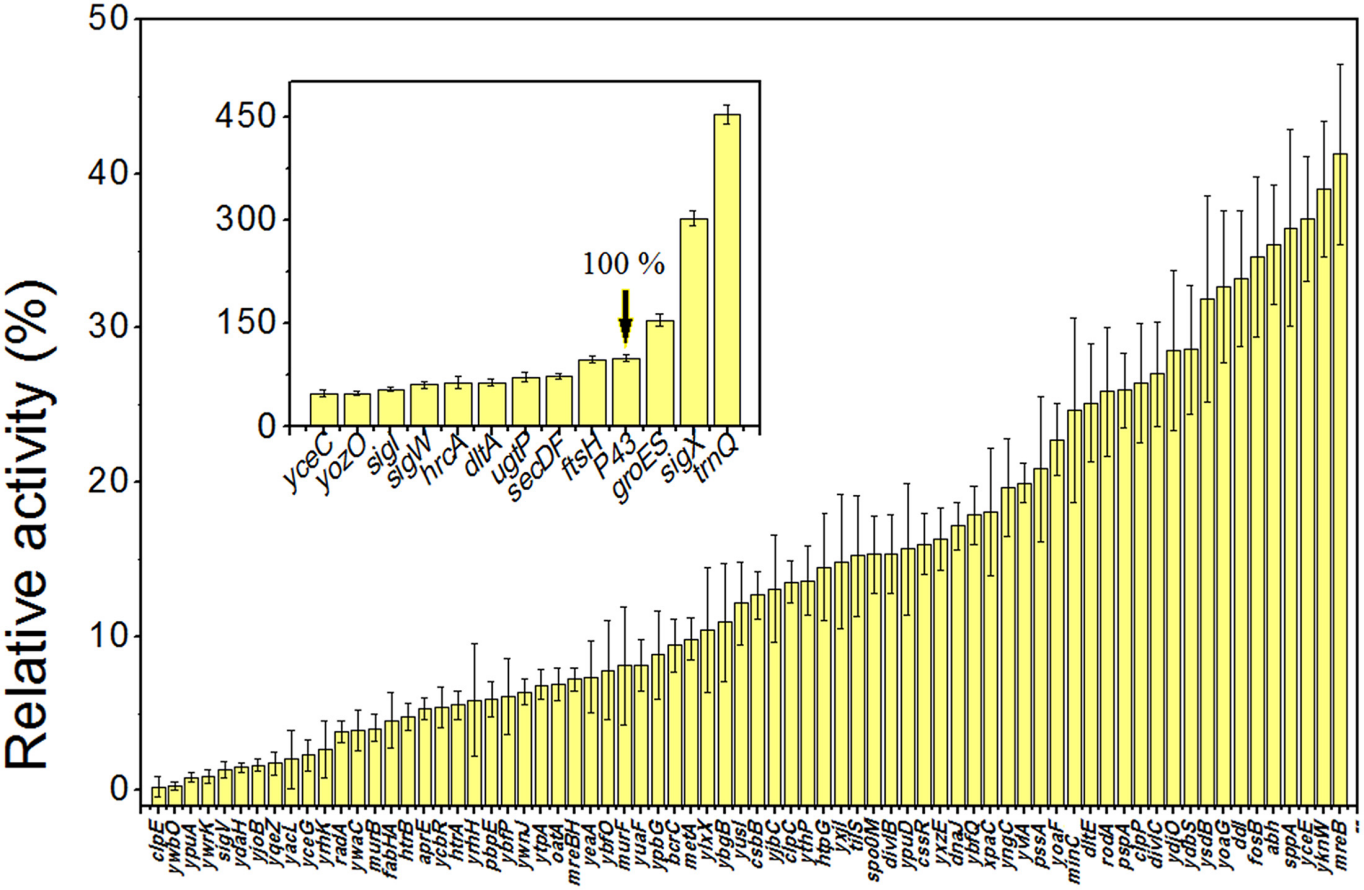
Strength of different promoter candidates measured by microplate reader. The relative expression level is the activity of each promoter compared to the strong constitute promoter P43. The activities of most promoters were less than 50% the activity of P43. Promoters with activities greater than 50% P43 were shown in the inset histogram, and P43 activity was set as 100%. Data are means ± standard deviation for three independent experiments.

PCR fragments (2 kb) corresponding to the sequences upstream of 84 genes were amplified with the oligos shown in S1 Table and, after digestion with the enzymes indicated, inserted into the pDL-GFP vector. These 84 were chosen because they were candidates to be expressed by specialized sigma factors (S2 Table) and/or under stress conditions (S3 Table). The promoter-*gfp* constructs were subsequently inserted into the *B*. *subtilis* chromosome at the *amyE* locus in single copy. The strengths of the cloned promoters were measured in a multimode microplate reader and the RFU of GFP signal from different promoters ranged from 0.0023 to 4.53 when compared to GFP fluorescence promoted by P43 (Fig 2). When we calculated the relative strengths of these promoter candidates, background expression level of promoterless-gfp was subtracted. Of these promoter candidates, P*trnQ* was more than four-fold stronger than the P43 promoter, used as a strong reference promoter. *trnQ* codes for arginine-specific tRNA. P*sigX* and P*groES* exhibited 3.03 and 1.55 times of P43’s strength in expression, respectively. In addition, P*secDF* and P*ugtP* showed slightly lower strengths than P43. There were also many promoters presenting low activities, many of them upstream of hypothetical proteins with unknown functions.

### Sequence analysis of strong promoters from *B*. *subtilis*

**Fig 3:**
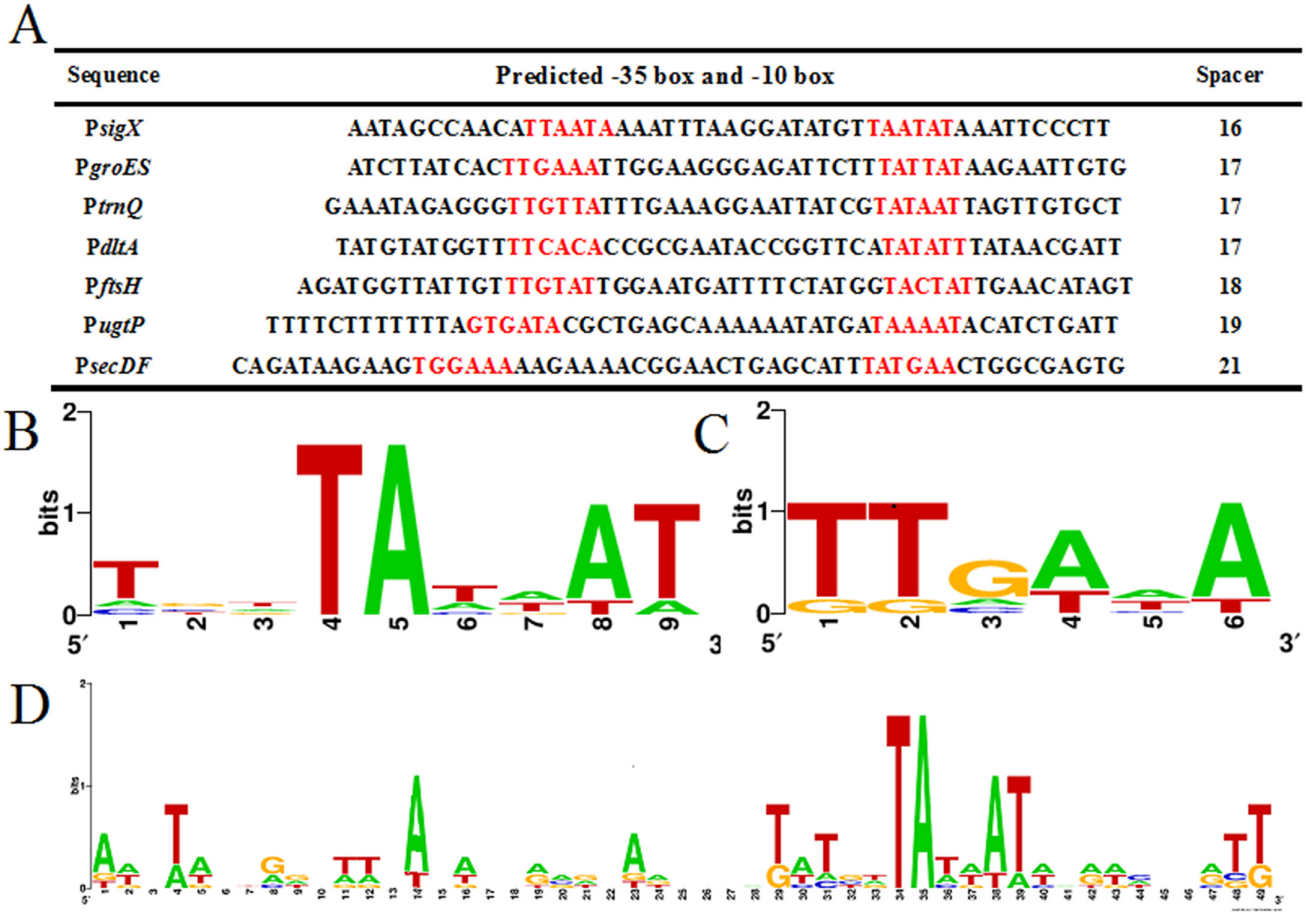
Sequence logo of the seven relatively strong promoter candidates from *B*. *subtilis*. A: Sequences of predicted -35 and -10 boxes and the spacers between the two elements. About half of the spacers between -35 and -10 boxes were around 17 base pairs. These three sequence logos were constructed using Weblogo3. B: Sequence logo of -10 hexamers; similar to cardinal “TATAAT” sequence. C: Sequence logo of -35 hexamer, not consistent with the classical “TTGACA” sequences. The -35 logo was very consistent with the consensus logo (5/6 positions were majoritarily correct). This was better than the -10 logo where only 4/6 positions were mostly the consensus base. D: Sequence logo of promoter candidates when the -10 box was fixed; the different spacing between the promoter elements may have contributed to difficulty in assigning the -35 box. This was presumably because the best-fit -10 sequences were used to make the logo. Since the varying lengths of the spacers have not been taken into account, there was no consensus -35 box.

We selected seven strongest promoter candidates and predicted the -35, -10 elements and the lengths of the spacers (Fig 3A) using Softberry Inc., (http://linux1.softberry.com/berry.phtml?topic=bprom&group=programs&subgroup=gfindb). These promoter candidates exhibited conserved -10 boxes, which were similar to the classical “TATAAT” sequence (Fig 3B), which was determined by applying Weblogo (http://weblogo.berkeley.edu/) [38,39]. However, the -35 sequences from these promoters were not consistent with the classical “TTGACA” (Fig 3C). Only 3 of the 7 promoters showed a canonical 17 bp spacer region. The differences in the lengths of the spacers could explain why we did not obtain obvious conserved sequences at particular positions, *e*. *g*. the -35 boxes, when we fixed the -10 boxes (Fig 3D). Additionally, the predicted -10 and -35 elements did not always match the experimentally validated actual -10 and -35 boxes. Use of another software tool for the same prediction gave varied results. An alternative program predicted promoters in the same region without precisely identifying -35 and -10 elements (results not shown). The predicted -10 and -35 boxes are listed in S2 Table as well as their regulated sigma factors. In *B*. *subtilis*, RNA polymerase consists of αα’ββ’ and one of several σ initiation factors which determines the specificity of RNA polymerase [40]. σ^A^ regulates the largest number of operons and is the primary “housekeeping” sigma factor, and the alternative sigma factor σ^B^ is activated in response to general stress or energy depletion [41]. σ^F^, σ^E^, σ^G^, and σ^K^ play important roles in sporulation [42]. Among extracytoplasmic function (ECF) sigma factors, σ^M^, σ^W^, σ^X^, and σ^V^ confer resistant against cell envelope active compounds, and σ^M^, σ^W^, σ^X^, σ^Y^, and σ^V^ control cell envelope stress proteins [43]. In addition, some genes had multiple promoters and overlapping recognition was observed due to the similar promoter recognition properties of some of these ECF sigma factors.

### Fluorescence-activated cell sorter (FACS) analysis of promoter candidates from *B*. *subtilis*

**Fig 4:**
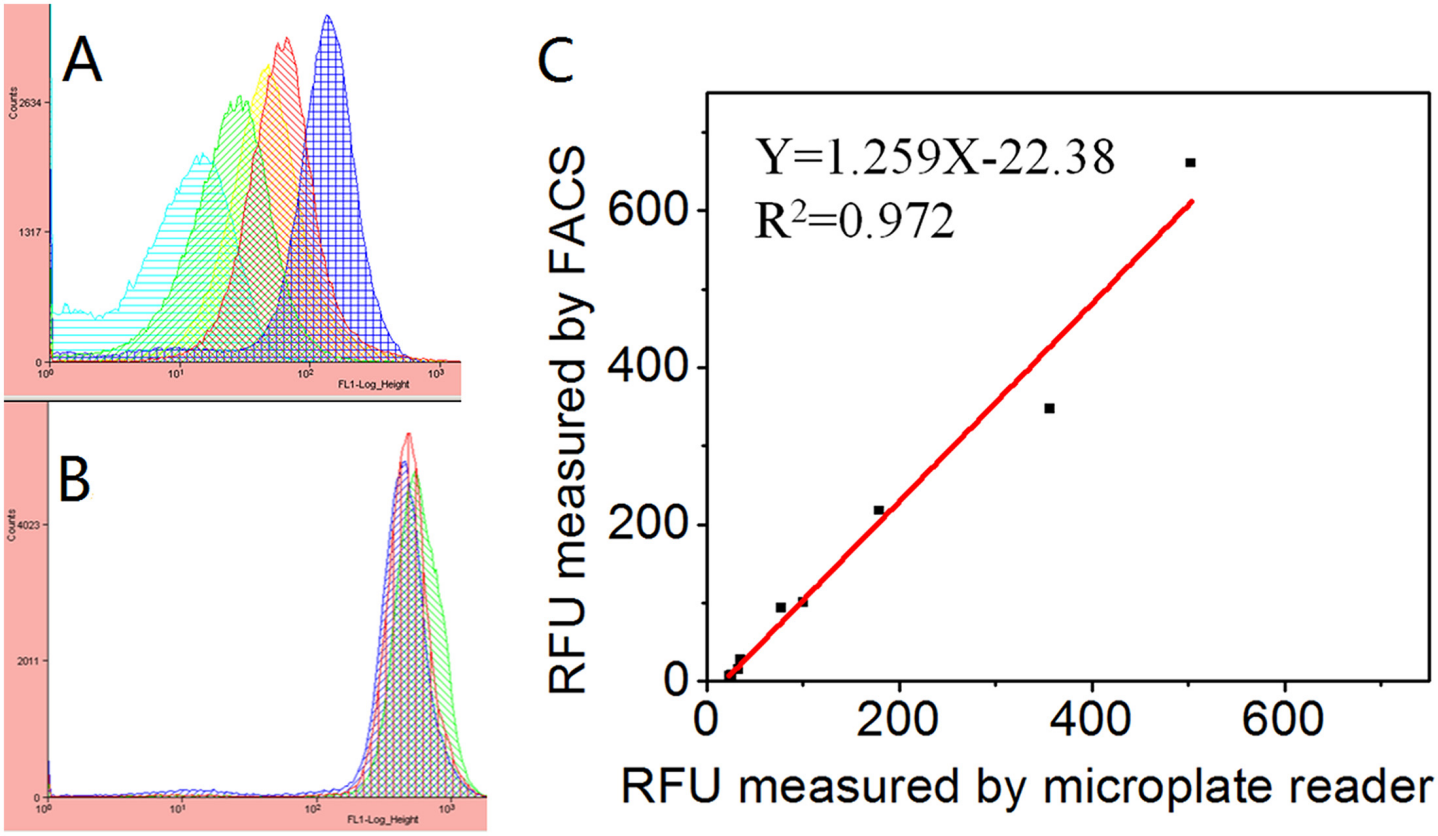
Comparison of promoter strengths from the two measuring methods. The X and Y axis represent results from microplate reader and fluorescence-activated cell sorter (FACS) analysis, respectively. A: Transformants of different promoter candidates showed different results. B: Three transformants of the same promoter candidates showed similar results. C: Relationship between different promoter candidates’ strengths measured by microplate reader and FACS.

FACS analysis was performed on bacteria growing in the logarithmic phase. Data analysis confirmed the fluorescence of GFP, positioned downstream of 10 different promoter candidates. Selected promoter candidates activated transcription at high, medium, and low levels. There was a good correlation between the data generated by the microplate reader and the FACS analysis, confirming the relative strengths of the candidate promoters (Fig 4).

### Changes in transcriptional level for different strains under stress conditions

**Fig 5:**
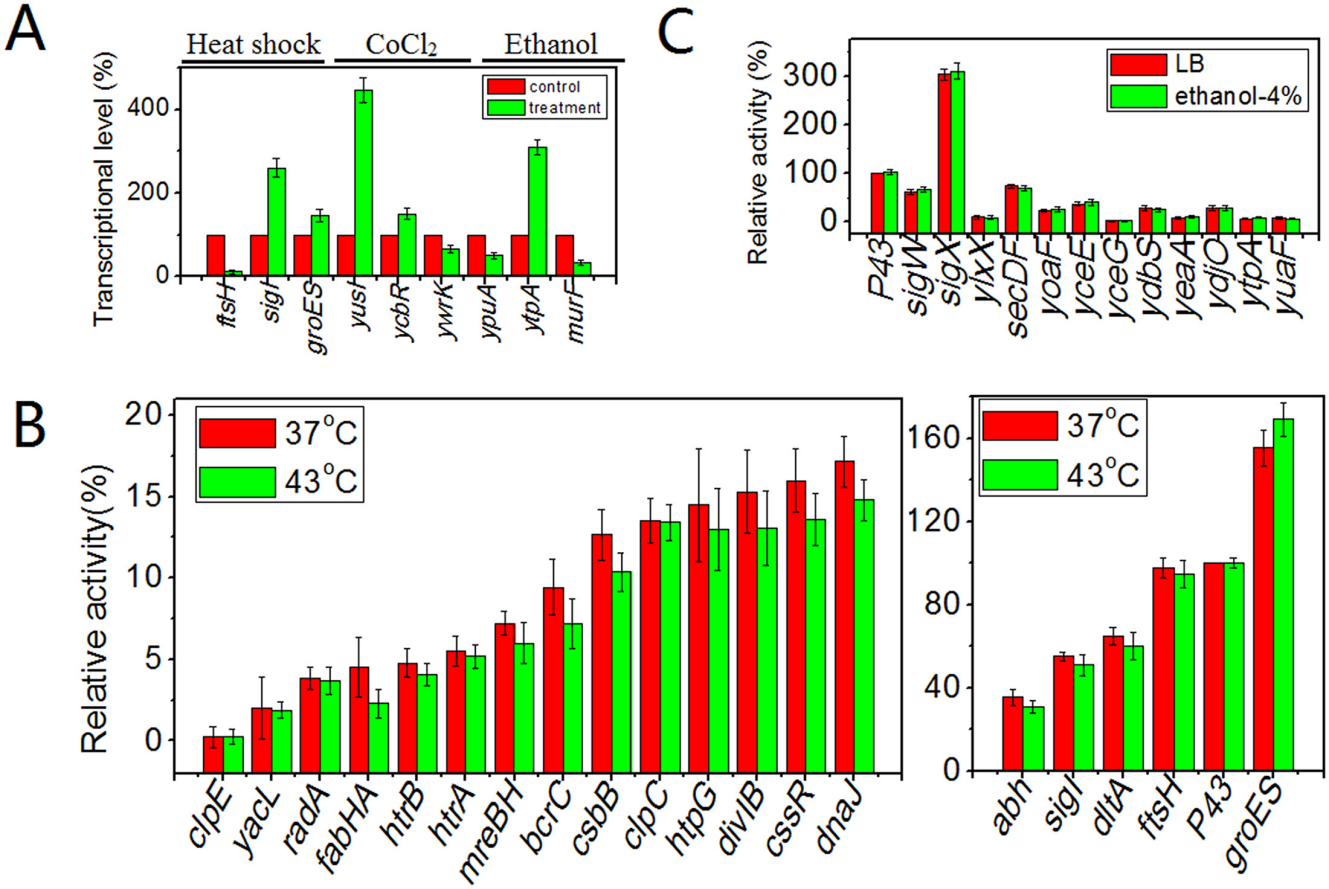
Changes of activity of promoter candidates at the transcriptional and expression level under different conditions. A: Level of changes of mRNA level of GFP measured by real-time fluorescence quantitative PCR after specific condition treatments corresponding to equivalent cultures incubated in LB media at 37°C. Strains carrying promoter candidates P*sigI*, P*groES*, P*ftsH* were transferred to 43°C for 15 min after incubating at 37°C for 4.5 h. Strains carrying promoter candidates P*yupA*, P*ytpA*, P*murF* were incubated in LB media with 4% (v/v) ethanol for 15 min after incubating in LB media for 4.5 h. Strains carrying promoter candidates P*yusI*, P*ycbR* and P*ywrK* were incubated in LB media with 8 mM CoCl_2_ for 15 min after culturing in LB media for 4.5 h. B: Relative activity of GFP expression level of different promoter candidates when cultured in LB media at 37°C for 5.5 h and heat shock at 43°C for another one hour. C: Relative activity of GFP expression level of different promoter candidates when cultured in LB media and LB media with 4% (v/v) ethanol for 6.5 h.

Since many of these predicted promoters were originally located in front of heat shock proteins (P*sigI*, P*groES*, and P*ftsH*) and proteins that respond to stress conditions (P*yusI*, P*ywrK*, P*ycbR*, Py*puA*, Py*tpA*, and P*murF*), we speculated that their expression would increase after the appropriate stress treatments. First, we measured *gfp* transcriptional levels by real-time fluorescence quantitative PCR for 9 different promoter candidates after treatments, such as heat-shock or high osmotic pressure treatments. Bacteria were cultured in 5 mL of LB media at 37°C for 4.5 h, and then subject to stress conditions. P*sigI*, P*groES*, and P*ftsH* cultures were subject to 15 min of heat shock at 43°C. This sudden temperature upshock is sufficient to induce the synthesis of heat shock proteins including both molecular chaperones such as GroES and ATP-dependent protease such as FtsH [44]. *sigI* encodes the RNA polymerase sigma factor σ^I^, and is required for growth at higher temperatures [45]. Cell envelope stress can result from chemical or genetic impairment, and chemical stress may include salt, ethanol, and superoxide [46]. Cultures of P*ypuA*, P*ytpA* and P*murF* in LB were supplemented with ethanol at a final concentration of 4% (v/v) for 15 min. *yupA* encodes a hypothetical protein, and its promoter was previously reported to be responsive to increases in the stress-responsive extracytoplasmic function ECF sigma factor sigM [47]. *ytpA* encodes a protein homologous to lysophospholipase, and sporulation was impaired when *ytpA* was blocked [48]. *murF* encodes UDP-N-acetylmuramoyl-tripeptide—D-alanyl-D-alanine ligase, which is responsible for the last step in the synthesis of cell wall peptidoglycan. P*yusI*, P*ycbR*, P*ywrK* were grown and treated with CoCl_2_ to a final concentration of 8 mM for 15 min, since proteins encoded by *yusI*, *ycbR and ywrk* were predicted to confer resistance to toxic metals, based on similarity. *ycbR* and *yusI* encode proteins that are similar to toxic cation resistance protein and arsenate reductase [49], respectively. *ywrK* encodes an arsenical membrane pump. There were rapid alterations in transcription levels after the treatments, confirmed by qRT-PCR results (Fig 5A). Surprisingly, P*ftsH* and P*murF* levels decreased dramatically, P*ywrK* and P*ypuA* were slightly reduced, and the other promoters showed increased activities. We next measured the expression levels of *gfp* after these stress treatments.

### Strength of *B*. *subtilis* promoter candidates under different stress conditions

We measured the transcriptional levels as the first step to assess potential activity changes for the specific promoters under different conditions. Despite appreciable changes, both increases and decreases, in mRNA levels (as measured by qRT-PCR) (Fig 5A), we did not detect significant changes in GFP activity (S1B Fig). We present the comparison of changes of selected promoters for both transcription and protein expression after the different treatments in S1 Fig. We assumed that after stress treatments, tested promoter candidates would respond by increasing expression level. Unexpectedly, some of these heat shock proteins’ promoters exhibited decreased but not increased activities in driving *gfp* expression when incubated under the stress conditions of 48°C or 43°C for 6.5 h (Data not shown). This may be because too long time at this high temperature may inhibit vegetative growth. In particular, the temperature treatment of 48°C caused a majority of bacteria to show a slow-growth phenotype. Necessary enzymes involved in central carbon metabolic and cell division might be inhibited under this stress. If the time for heat shock was short, *e*.*g*. 15 min, there were sharp shifts in transcriptional levels detected through qRT-PCR (Fig 5A). However, longer time (*e*.*g*. one hour) was essential to allow accumulation of proteins to test the response to heat shock (Fig 5B). However, after incubation at 37°C for 5.5 h and heat shock at 43°C for another one hour, any changes in GFP expression were minor (Fig 5B) compared to the mRNA changes measured for the subset of 9 promoters studied by qRT-PCR (Fig 5A). The heat shock stimulon is quite complex, and involves several classes of heat shock genes controlled by transcriptional repressors, alternative sigma factors, and two-component signal transduction system. Different temperatures, heat shock periods, growth media and strains may influence the final results [44].

**Fig 6:**
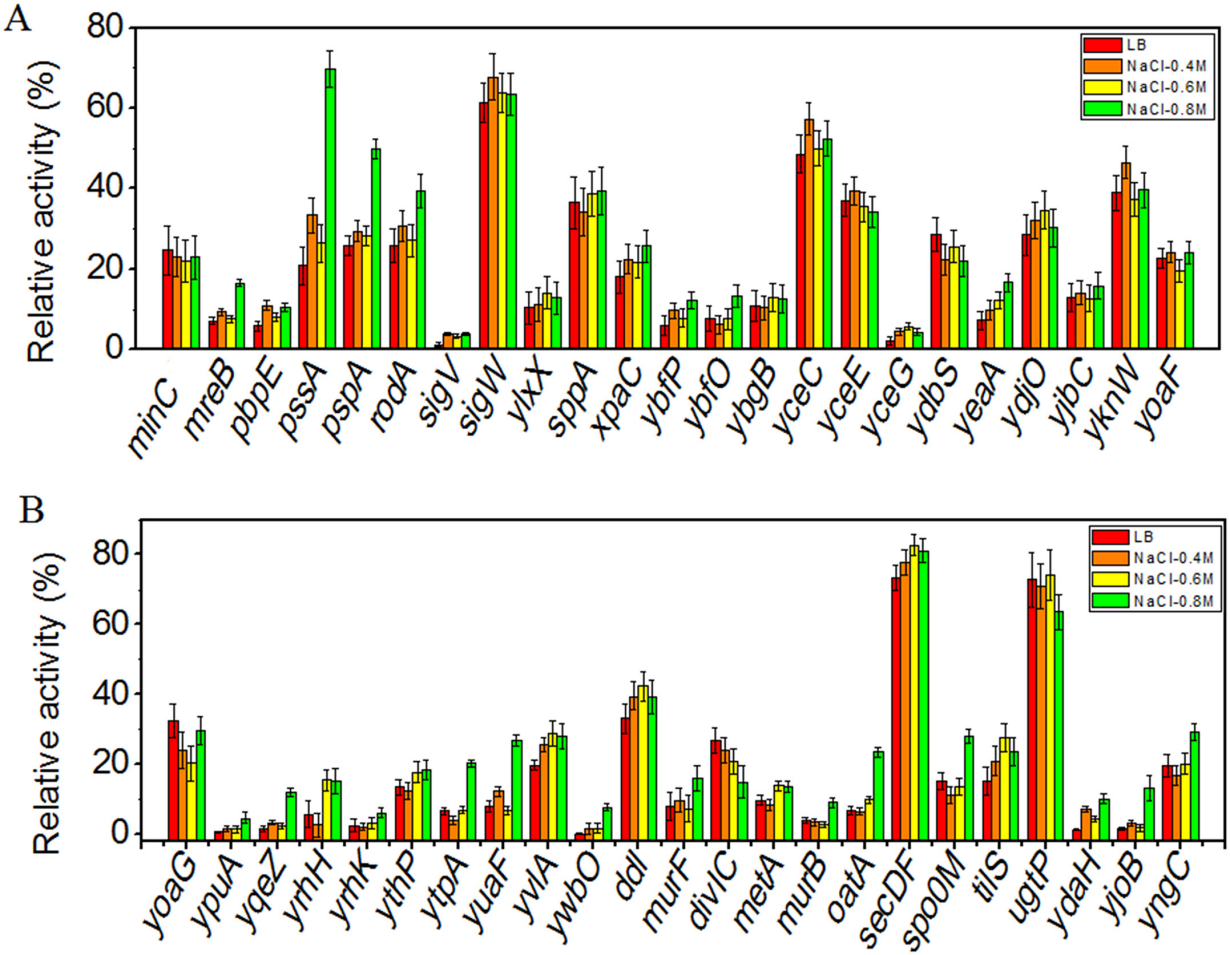
Strength of different promoter candidates under different conditions. The strength of promoters was measured when strains were cultured in LB media alone, or supplemented with 0.4 to 0.8 M NaCl for 6.5 h. Only some promoters showed a slight response with 0.8 M NaCl. Error bars represent standard deviations of biological triplicates. Activities of most promoter candidates did not change relative to the activities in LB.

Next, we investigated whether promoter candidates respond to stress by changing the growth conditions. For this, we inoculated these bacteria into LB media containing 4%, 6%, or 8% (v/v) ethanol. At ethanol concentrations of 6% and 8%, bacterial growth was slowed. However, we detected no significant changes in GFP expression after treatment with 4% ethanol (Fig 5C). 11 of 46 promoter candidates showed a relatively sharp response after incubation in LB with 0.8 M NaCl (Fig 6). However, of those, strains carrying P*mreB*, P*pssA*, P*oatA*, P*ytpA*, P*yuaF*, P*ydaH*, P*yjoB*, and P*ywbO* showed no changes in expression at 0.4 M or 0.6 M NaCl (Fig 6). At final concentrations of 1.2 M NaCl and 6.5 h of incubation, bacterial growth was inhibited.

### Correlation between BgaB activity and GFP fluorescence for promoter candidates

**Fig 7:**
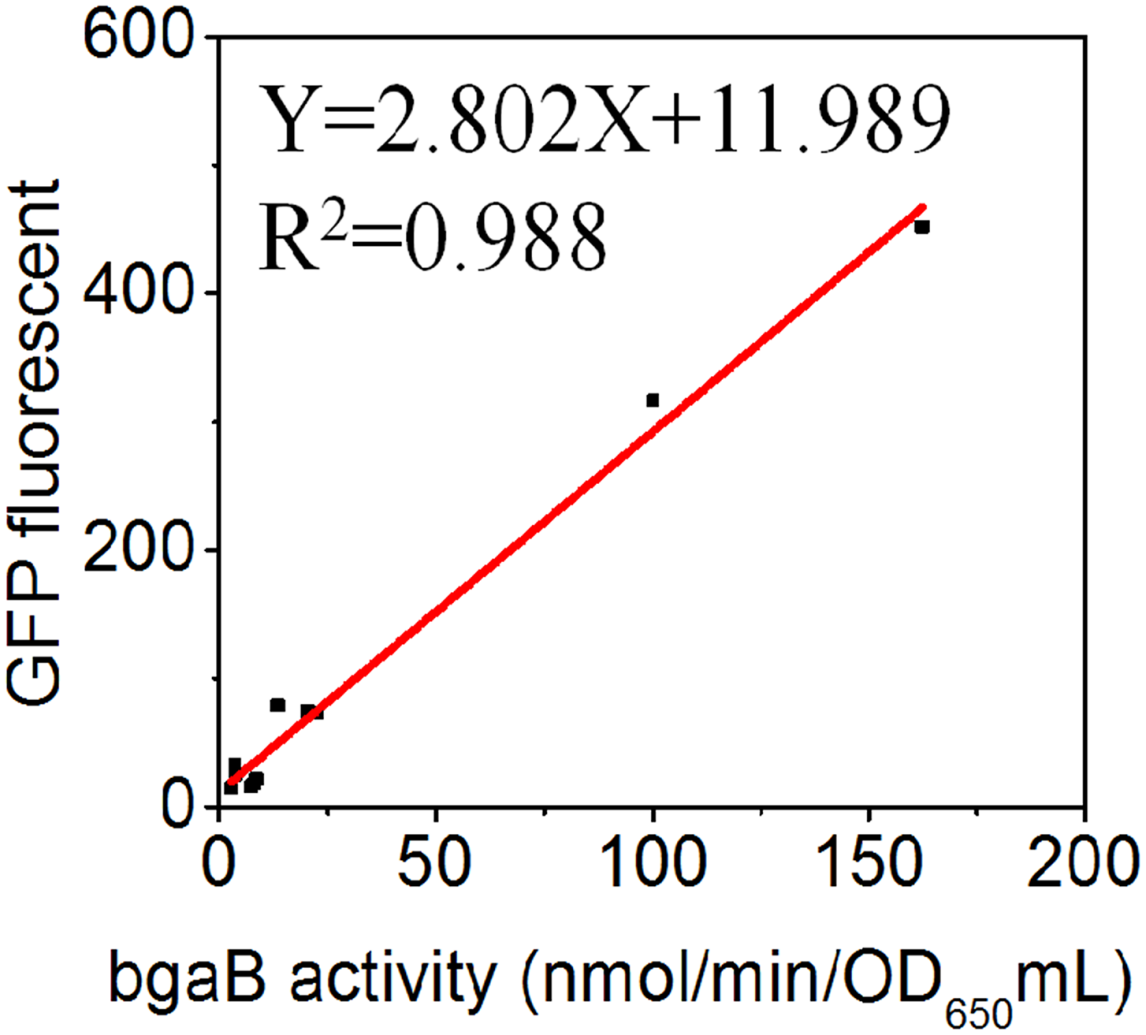
Relationship of thermostable β-galactosidase (BgaB) and GFP expression levels. The same promoter candidates were tested upstream of β-galactosidase (BgaB) and GFP and showed similar results. ONP was detected at OD_420_ indicating that ONPG, the substrate of BgaB, was hydrolyzed.

It is important that activities of promoters we measured are context-independent, i. e., that promoter activities remain constant regardless of the downstream gene being regulated [50]. To investigate this, we compared the GFP activity from plasmid pDL-GFP with that of the same promoter in the original plasmid pDL, with the reporter gene, bgaB, encoding thermostable β-galactosidase. We selected 10 promoter candidates spanning a wide range of strengths, including P*radA*, P*mreBH*, P*murF*, P*ywnJ*, P*yxjI*, P*yxzE*, P*trnQ*, P*sigX*, P*secDF*, and P*ugtP*. Except for the different promoter fragments, the backbone of pDL carrying *bgaB* was the same as the one carrying *gfp*. There was a linear correlation between the measured GFP and BgaB activities (Fig 7). Thus, the effects we tested were independent of the reporter gene.

### Increased secretion production of α-amylase in *B*. *subtilis*

**Fig 8:**
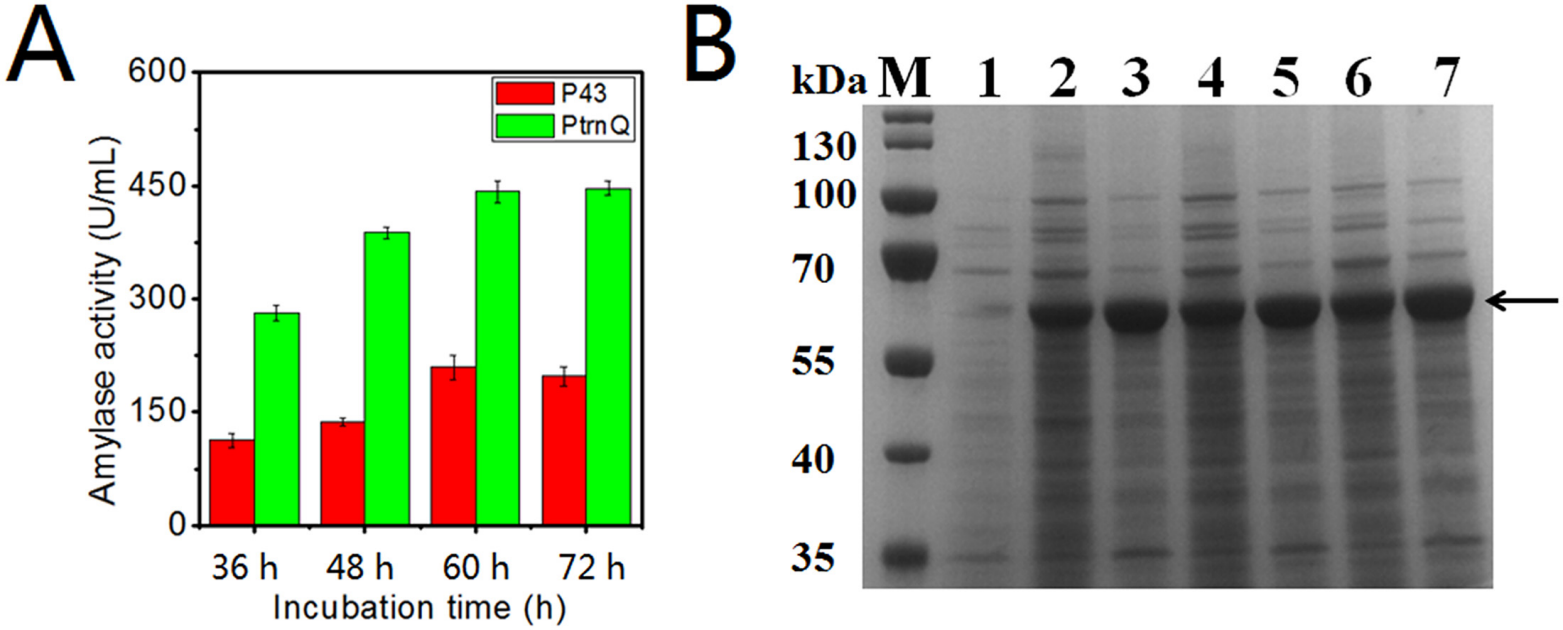
Comparison of P43 and P*trnQ* driving α-amylase in pMA5. A: Enzyme activity of α-amylase that secreted into the medium during fermentation in flasks for 72 h. Error bars represent standard deviations of biological triplicates. B: SDS-PAGE analysis of α-amylase in the supernatant secreted by *B*. *subtilis* 1A751. 10 μL of each supernatant sample was loaded on the gel. Lane M: molecular weight marker; Lane 1: pMA5 without α-amylase; Lane 2, 4, 6 represented pMA5 containing P43 after incubation for 36 h, 48 h, and 60 h, respectively. Lane 3, 5, 7 represent pMA5 containing P*trnQ* after incubation for 36 h, 48 h, and 60 h, respectively.

To further validate the promoter activities, we substituted the P43 promoter upstream of the secreted protein α-amylase with P*trnQ* in the pMA5 plasmid. *B*. *subtilis* 1A751 was chosen as the host strain for the expression of this shuttle-plasmid. During 72 h of fermentation in 250 mL triangular flasks, α-amylase was secreted into media and the activity was analyzed every 12 h. The enzyme activity peak time occurred close to 60 h. The α-amylase production driven by P*trnQ* was only twice as high as that from the P43 promoter. This is less than the fourfold observed when *gfp* and *bgaB* were used to monitor P*trnQ* and P43 activities (Figs 2 and 8). The difference could be because α-amylase is a secretory protein and some secretion bottlenecks might hinder the efficient transportation of this protein. However, despite the differences in the fold activation, this result showed that P*trnQ* is a strong promoter compared to P43 and could be used to improve the production of secreted proteins.

## Discussion

Promoters are fundamental genetic materials that determine transcription and expression. The use of these elements as biobricks will allow their use in directed synthetic biology to obtain fine-tuned and balanced expression of genes. The Registry of Standard Biological Parts (RSBP) (http://parts.igem.org/Promoters) continues to collect an increasing number of genetic parts for use as components of biological devices and systems. However, until now, most promoter information has focused on sequences that work in *E*. *coli*. There have been some attempts to discover and optimize suitable promoters for *B*. *subtilis*, for instance, the Ludwig Maximilian University (LMU) of Munich team similarly tried to “open *Bacillus subtilis* and its unique biology to the iGEM world” by measuring promoter activity with reporter genes (http://2012.igem.org/Team:LMU-Munich/Data). However, only a small number of the promoters they used were native to *B*. *subtilis*. Other reports have tested individual specific promoters, but there has been no comprehensive analysis of different promoter candidates derived from diverse proteins. Hence, we determined to evaluate promoters of interest and normalize their strength to the well-characterized P43.

If genes are expressed at a high level in favorable, unfavorable or threatening circumstances, they are likely to contain strong promoters or promoters that may respond to certain environmental signals (http://science.sciencemag.org/content/suppl/2012/02/29/335.6072.1103.DC1). We selected 84 promoter sequences mainly located upstream of heat shock proteins, cell envelope proteins, and proteins resistant against toxic metals. Tested promoter candidates showed a range of strengths relative to P43, and several of these were tested for function in different genetic circuits, indicating their use in a variety of genetic contexts to allow gene fine-tuning. One approach is using pools of thousands of synthetic, codon-usage variants to try to regulate protein production, but this process is inefficient and expensive [51]. An alternative approach is to control metabolic pathways by precisely controlled gene transcription using validated promoters, particularly when only inefficient high-throughput screening methods are available. A successful example of the application of a promoter library is the genetic module created for the synthesis of fatty acid ethyl ester (FAEE) by dynamic sensor regulated systems in *E*. *coli* [52].

Prior to evaluation of these promoter candidates for their response to various stress treatments, we expected that some of the applied stresses would produce significant changes in gene expression. However, only 11 candidates (P*mreB*, P*pssA*, P*pspA*, P*spo0M*, P*yqeZ*, P*oatA*, P*ytpA*, P*yuaF*, P*ydaH*, P*yjoB* and P*ywbO*) exhibited appreciable shifts when incubating in LB with 0.8 M NaCl at 37°C for 6.5 h. In addition, the putative stress responsive promoters were actually weaker than the constitutive promoter P*trnQ* (Fig 2). Several reasons may explain this finding. Firstly, our small sample size may not have included many promoter candidates that are significantly responsive to these tested stress conditions continuously. Secondly, the profiles of mRNA and protein expression may not be strictly correlated, for example if significant mRNA processing occurs [53]. Thus, some promoter candidates may have responded to stress treatments by increasing their transcription levels, but that increased transcription may not have led to increased translation under these conditions [54]. Our experiment, Figs 5A and S1, showed this was the case for at least 9 of the promoters. Finally, during stress, in addition to changes of stress-specific protein expression levels, rearrangements in the composition and structure of cell envelope occur in order to obtain high salt-resistant capacity. This may affect genes involved with lipid and fatty acid composition of the cytoplasmic membrane [54]. For example, *ydaH* and *ytpA* encode lipid II flippase and phospholipase, respectively, proteins involved in lipid metabolism. Both YuaF and YqeZ belong to the NfeD family, and YuaF plays a role in maintaining membrane integrity during conditions of cellular stress [55]. In general, many promoters may maintain their relative activity levels under different growth conditions. The changes we observed, however, derived from global effects but not specific responses [56]. Before application of these so-called condition-inducible promoters, further validation was necessary to demonstrate specific regulated response rather than global regulation, as well as validating the specific sequences recognized by different sigma factors [57].

The promoter library constituted of intrinsic biological elements gives us a more clear understanding of their expression patterns when exposed to adverse culture conditions, which is beyond the reach of a synthetic promoter library. This can be a good complement to artificial promoters for synthetic biology and metabolic engineering. The promoter P*trnQ* was strong when positioned upstream of *gfp* and *bgaB*, but performed less well when we tried to improve the production of α-amylase. Though pMA5 contains an efficient native signal peptide of α-amylase SP_*amyl*_ [23], other bottlenecks such as a lack of chaperones may have limited the secretion efficiency of α-amylase. Overall, the yield of a secretory protein likely does not always depend linearly on promoter strength.

## Conclusions

We constructed an integrated plasmid to measure the activities of promoter candidates by using a *gfp* reporter. These promoter candidates were selected from four categories of proteins belonging to the group of heat shock proteins, stress response proteins, or proteins which are resistant against toxic metals (based on similarity) and other kind of genes. We compared these 84 promoter candidates to the well-characterized P43 to determine the promoters’ relative strength. Tested promoters can now be considered for use as biobrick elements in *B*. *subtilis*. One strong promoter, P*trnQ*, increased the secretion of α-amylase to 2.1 fold that of P43 driven expression. Other inducible promoters will be useful for fundamental research or application in industrial production.

## Supporting Information

S1 Fig Comparison of changes of GFP on the level of both transcription and protein expression after different stress treatments. A: Level of changes of mRNA level of GFP after specific condition treatments corresponding to equivalent cultures incubated in LB media at 37°C. Strains carrying promoter candidates P*sigI*, P*groES*, P*ftsH* were heated at 43°C for 15 min after incubating at 37°C for 4.5 h. Strains carrying promoter candidates P*yupA*, P*ytpA*, P*murF* were incubated in LB media with 4% (v/v) ethanol for 15 min after incubating in LB media for 4.5 h. Strains carrying promoter candidates P*yusI*, P*ycbR* and P*ywrK* were incubated in LB media with 8 mM CoCl_2_ for 15 min after culturing in LB media for 4.5 h. B: Level of changes of expression level of GFP after specific condition treatments corresponding to equivalent cultures incubated in LB media at 37°C. Strains carrying promoter candidates P*sigI*, P*groES*, P*ftsH* were heated at 43°C for 1 hour after incubating at 37°C for 5.5 h. Strains carrying promoter candidates P*yupA*, P*ytpA*, P*murF* were incubated in LB media with 4% (v/v) ethanol for 6.5 h. Strains carrying promoter candidates P*yusI*, P*ycbR* and P*ywrK* were incubated in LB media with 8 mM CoCl_2_ for 1 hour after culturing in LB media for 5.5 h.
(TIF)

S1 Table Primers used to amplify promoter candidates in this study. (DOCX)

S2 Table Predicted -10 boxes, -35 boxes, spacers and regulated sigma factors of these promoter candidates. (DOCX)

S3 Table Functions of proteins encoded by genes downstream of these promoter candidates and their categories. (DOCX)
