# Supplementary figures and images for: Promoter Screening from Bacillus subtilis in Various Conditions Hunting for Synthetic Biology and Industrial Applications
Source: PLoS One. 2016 Jul 5;11(7):e0158447. doi: 10.1371/journal.pone.0158447 (PMC4933340; doi:10.1371/journal.pone.0158447)

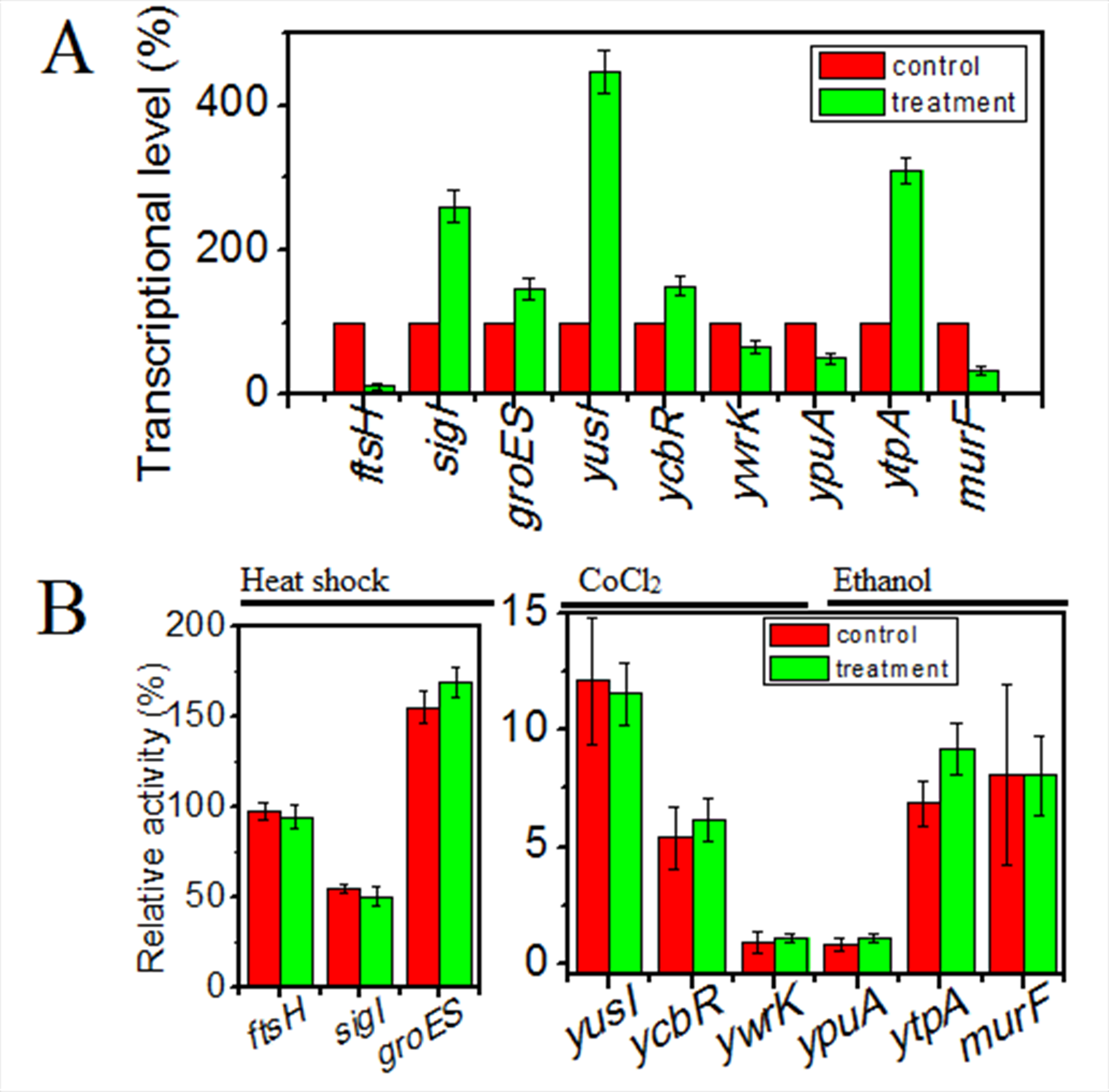

Supplement: S1 Fig — A: Level of changes of mRNA level of GFP after specific condition treatments corresponding to equivalent cultures incubated in LB media at 37°C. Strains carrying promoter candidates PsigI, PgroES, PftsH were heated at 43°C for 15 min after incubating at 37°C for 4.5 h. Strains carrying promoter candidates PyupA, PytpA, PmurF were incubated in LB media with 4% (v/v) ethanol for 15 min after incubating in LB media for 4.5 h. Strains carrying promoter candidates PyusI, PycbR and PywrK were incubated in LB media with 8 mM CoCl2 for 15 min after culturing in LB media for 4.5 h. B: Level of changes of expression level of GFP after specific condition treatments corresponding to equivalent cultures incubated in LB media at 37°C. Strains carrying promoter candidates PsigI, PgroES, PftsH were heated at 43°C for 1 hour after incubating at 37°C for 5.5 h. Strains carrying promoter candidates PyupA, PytpA, PmurF were incubated in LB media with 4% (v/v) ethanol for 6.5 h. Strains carrying promoter candidates PyusI, PycbR and PywrK were incubated in LB media with 8 mM CoCl2 for 1 hour after culturing in LB media for 5.5 h. (TIF) [file pone.0158447.s001.tif]
