# Supplementary material for: Promoter Screening from Bacillus subtilis in Various Conditions Hunting for Synthetic Biology and Industrial Applications
Source: PLoS One. 2016 Jul 5;11(7):e0158447. doi: 10.1371/journal.pone.0158447 (PMC4933340; doi:10.1371/journal.pone.0158447)
Supplement: S1 Table — (DOCX) [file pone.0158447.s002.docx]

**S1 Table. Primers used to amplify promoter candidates in this study.**

| Name | Sequence |
| --- | --- |
| PclpcBam | gatcggatccgcctaaggtttcctgaggaagg |
| PclpcKpnI | gatcggtacctcaaccccctcctttactgact |
| PclpeBam | gatcggatcccgaatgtggatggcagttcgcc |
| PclpeKpnI | gtacggtaccttgccaaaacctccttaataat |
| PcssrBam | gatcggatcctatgaccatgcggctgaaacag |
| PcssrKpnI | gatcggtaccggctcttcacatcctttcaacg |
| PclppEcoR | gatcgaattctagccggggtctcctttgaaga |
| PclppBam | gatcggatccaatgctcctccttcaccttttag |
| PradaBam | gatcggatccggaagacagccttgaccctgta |
| PradaKpnI | gatcggtaccatatagtgtaagacctctcccttg |
| PyaclBam | gatcggatccggtcagagaatgtaccgctgag |
| PyaclKpnI | gatcggtaccacccccacctccttttttaaca |
| PdnajBam | gatcggatccggaacaacaaactcatgtgtgg |
| PdnajKpnI | gatcggtaccctcgcttcactctcccgaattt |
| PftshBam | gatcggatccacaatcattgtaggcgtttccg |
| PftshKpnI | gatcggtacctccttacctcctcccacagtaag |
| PgroesBam | gatcggatccgaagatcagtaagttaggatgacacg |
| PgroesKpnI | gatcggtacctgaaataacctcctcaatagtatga |
| PhtpgBam | gatcggatcccggttttattggctgtttggaaa |
| PhtpgKpnI | gatcggtaccctcttgatcgctcctttaccatt |
| PhtrbBam | gatcggatcccccggcatcaaaatatcctctt |
| PtrnQBam | gatcggatccgatgcagtgggatgccggaaaac |
| PtrnQKpnI | gatcggtaccatatagactgcgttatgagaacgtcag |
| PhtrbKpnI | gatcggtaccgttcttacactcctttaacggt |
| PmerbhBam | gatcggatcctgccttttccagcttcactttc |
| PmerbhKpnI | gatcggtaccgttttcacccaatctctatcct |
| PsigiBam | gatcggatcccatgaaacaagggagttggtgg |
| PsigiKpnI | gatcggtaccctcagttcctccctataactactc |
| PbrccBam | gatcggatccgtcaattcccttttcagctcat |
| PbrccKpnI | gatcggtaccataatcaccttttacatttttatattta |
| PhtraBam | gatcggatccaaaacgctttatcatgtatcgcg |
| PhtraKpnI | gatcggtacccatgttcactccgtttctctat |
| PycbrBam | gatcggatcctgcgtaaaggaggagagacacg |
| PycbrKpnI | gatcggtacctctgtcatctccgtcagctctt |
| PyusiBam | gatcggatcctgaaatgaaacggagaaacgagca |
| PyusiKpnI | gatcggtacccttcttccccctcaataaaataatcg |
| PywrkBam | gatcggatcccaaatggaagctgaacctttttc |
| PywrkKpnI | gatcggtacctgtgaatatgccggtgaactcaa |
| PabhBam | gatcggatccccgcaatccgtttggaatctata |
| PabhKpnI | gatcggtaccaaaaacccttcttcctttaaatgtttct |
| PcsbbBam | gatcggatccgtaatgctcgctttaacagctg |
| PcsbbKpnI | gatcggtacctaaggcaccttctttttattattcttt |
| PdivibBam | gatcggatccgtcaagactgtgatgcgttttctg |
| PdivibKpnI | gatcggtacctgcctgttcacctcattcaaatg |
| PdltaEcoR | gatcgaattctaaaggaacagatgatgcgcac |
| PdltaKpnI | gatcggtaccagttattctctctccaattagaaatcg |
| PfabhaBam | gatcggatccgtatatgaagaagaccgaaaagaatgg |
| PfabhaKpnI | gatcggtacctagggaagactcctttatatcttaaat |
| PfosbBam | gatcggatccacaggaaaggaagggtttatgg |
| PfosbKpnI | gatcggtaccaaatcattccccccttgaaagc |
| PmincKpnI | gatcggtaccaatattcacctcaacaacatactcatttc |
| PmincBam | gatcggatccgtacgacagaagttgcgattatttcc |
| PpssaBam | gatcggatccggacggaaagcaaagacaccaa |
| PpssaKpnI | gatcggtaccagtgtaaccaaacctccaattgt |
| PpspaEcoR | gatcgaattcgtgatttcgagcatgagctttattaa |
| PpspaKpnI | gatcggtacctatatcgttcctcctaaagctaattcgg |
| ProdaEcoR | gatcgaattctgattcagttgaagaaacgc |
| ProdaKpnI | gatcggtacctctatcccgccttacattttcatc |
| PsigvBam | gatcggatcctgatacacgtattgctttgtttgtc |
| PsigvKpnI | gatcggtacctgcaataaagggctcctttttgaat |
| PsigwBam | gatcggatcccgttgttgatcattttaaggatgc |
| PsigwKpn | gatcggtaccatttatctaacctctgccttcacc |
| PsigxBam | gatcggatccaggttataaatttgaggtcggcg |
| PsigxKpn | gatcggtaccttgaaacccctccgttcacttt |
| PylxxEcoR | gatcgaattctgttttgcagcttgagcagaag |
| PylxxKpnI | gatcggtacctctatttcacccgctttttcagt |
| PsppaEcoR | gatcgaattcatgaggcattaacggctgcaataaa |
| PsppaKpnI | gatcggtacctttttctcctcctttttcctaaactctc |
| PxpacBam | gatcggatccgcgaggagagaatcctaaggtg |
| PxpacKpnI | gatcggtaccgagataatcgactcctgtgtct |
| PybfpBam | gatcggatcctcatttggctcaaccgtcattt |
| PybfpKpnI | gatcggtacctgcacgctccaatctcctttct |
| PybfoBam | gatcggatcccttacagtgtcgcgttgtatacc |
| PybfoKpnI | gatcggtaccttctatctctccttttctgctaggttc |
| PybgbBam | gatcggatccatattgtcgagggtgacggtta |
| PybgbKpnI | gatcggtaccagaaccaccattcccttcttaaaac |
| PycecEcoR | gatcgaattcttttttctcagcaccaatataaccg |
| PycecBam | gatcggatccacgattcactcctactcatcaaaa |
| PyceeEcoR | gatcgaattcctttgattccggcatacggatg |
| PyceeBam | gatcggatccactgcctctctcctttctgc |
| PycegEcoR | gatcgaattcatcttgactcaagtgtgtttctgt |
| PycegBam | gatcggatcccatatcctcctttccggtttgt |
| PydbsEcoR | gatcgaattcactaatgtatgccggccgaaaa |
| PydbsKpnI | gatcggtaccaaatacctacctccctttttttcgtc |
| PyeaaBam | gatcggatccttccctttcctaacctacttgacc |
| PyeaaKpnI | gatcggtaccaaaaagcagctccttttgttgt |
| PydjoBam | gatcggatccataacccgacaaacttgcctct |
| PydjoKpnI | gatcggtaccaaagtcacttcttccctcatgt |
| PyjbcBam | gatcggatccacagtgggattgaacaaggaac |
| PyjbcKpnI | gatcggtacctgatttgctccttatgagttcgt |
| PyknwBam | gatcggatccgatttacgatttgcaataccgtg |
| PyknwKpnI | gatcggtaccttcaaaacctccttgagaatatctaac |
| PyoagBam | gatcggatccaatcgctcattatcattgtcacag |
| PyoagKpnI | gatcggtacctttttcaccccccttgtttgat |
| PyobjBam | gatcggatccaatcgataagctaaacaagcttg |
| PyobjKpnI | gatcggtaccttaagcttctccccttctctctg |
| PyozoBam | gatcggatcccgggtccgaaaatttcttctttt |
| PyozoKpnI | gatcggtacctgaagaacccctctttctgcat |
| PypuaBam | gatcggatcccctctgaataccgtccaaaaaaaag |
| PypuaKpnI | gatcggtaccagcagtttgacctccttcaatttc |
| PyqezBam | gatcggatccggacagtttgtccgtgtggttgagg |
| PyqezKpnI | gatcggtaccgggcggtgtatccctccttcct |
| PyrhhBam | gatcggatccgttatagaaggctaaagcaatggaatgt |
| PyrhhKpnI | gatcggtaccaactagacggcaatgtattg |
| PyrhkBam | gatcggatccgccgtcctgtcagttatcactt |
| PyrhkKpnI | gatcggtaccatcatccctccattcaattttggc |
| PythpBam | gatcggatccattgtcaggaccgacgtatacg |
| PythpKpnI | gatcggtaccaactcactcacctccaaaatttac |
| PytpaBam | gatcggatcctgattatttgggaccgcttgca |
| PytpaKpnI | gtacggtaccgatcttcacctcatacgaaatt |
| PyaufBam | gatcggatccgcacttgacaatcattgatcctg |
| PyaufKpnI | gatcggtacccttcctcatcctttcttcatcaatg |
| PyvlaBam | gatcggatccggctataaaaaaggccgtttcag |
| PyvlaKpnI | gatcggtaccttcattccacactcctattgtgaa |
| PywboBam | gatcggatccatcaagatatttccggggaaaa |
| PywboKpnI | gatcggtaccgataatctcctttcatactaaattgatttt |
| PddlBam | gatcggatcctggatgacgccgtgatacacaa |
| PddlKpnI | gatcggtacctaatcctagacttgttttcaatggatac |
| PmurfBam | gatcggatccttgatccggtaacggcttttat |
| PmurfKpnI | gatcggtaccgtaaggtccctctctttcttttgt |
| PdivicBam | gatcggatccagacgcatcagtcattaaagca |
| PdivicKpnI | gatcggtaccaccagacggtcctcctttca |
| PmetaBam | gatcggatcctctgacaccttgttctttccaag |
| PmetaKpnI | gatcggtacccgtgccacctccattatttccc |
| PmurbBam | gatcggatccagtttatgaagcttgcgatgattg |
| PmurbKpnI | gatcggtacccgtaaacctccgcattccattt |
| PoataBam | gatcggatccgtacggagacatcgtatctgatt |
| PoataKpnI | gatcggtaccgggtattcctccaatagtatgca |
| PsecdfBam | gatcggatccatgatcaaccgtccgataatgg |
| PsecdfKpnI | gatcggtacctatgtatatcctcccttaaacctgtgc |
| PspoomBam | gatcggatccggagcggctttaatccttcttgaata |
| PspoomKpnI | gatcggtaccgttagatcttcccctttttcttttttc |
| PtilsEcoR | gatcgaattcttgttgtcgtccggattgatca |
| PtilsKpnI | gatcggtaccaatgtcctcctcacaatgagca |
| PugtpBam | gatcggatccctctgcctgcttcaattccttc |
| PugtpKpnI | gatcggtaccgtaaattcacctcaatgtaatcaacaaca |
| PydahBam | gatcggatcctacagaccgttattgttcaaagg |
| PydahKpnI | gatcggtaccgttattcttcctccaatttcatcct |
| PyjobBam | gatcggatccggtgaagtactcaagacaaaaggg |
| PyjobKpnI | gatcggtaccgtttatactccatttcatgttttgtg |
| PyngcBam | gatcggatcccctttttccttaaaatggggga |
| PyngcKpnI | gatcggtaccattcttcacaacctgtcctaatct |
| PyoafBam | gatcggatccggagaccctgatcatcctgtca |
| PyoafKpnI | gatcggtaccgtgtatgcctccattatgtcgatg |
| PypbgBam | gatcggatccccttgtatgaacaaaaagggga |
| PypbgKpnI | gatcggtaccaaaaaactctccattctttttagaact |
| PypudBam | gatcggatccgttctaacttttttttggacggtattc |
| PypudKpnI | gatcggtaccttctaaacctccagccaatatgaat |
| PysdbBam | gatcggatccttatcgaatgtcagctccggat |
| PysdbKpnI | gatcggtacctgtgtgaaggcacctcccttat |
| PywacEcoR | gatcgaattccgataattttcgcaatccgttcg |
| PywacKpnI | gatcggtaccgttcgtcatctcctttaacgga |
| PywnjBam | gatcggatccatgatcccgatcaggacagtaac |
| PywnjKpnI | gatcggtaccgacataacctcctttataacgtacg |
| PyxjiBam | gatcggatccggatgcagccattgatgaaaac |
| PyxjiKpnI | gatcggtacctgctgaagtctcctttgggttt |
| PyxzeBam | gatcggatcctgacgttgccattttaagcgcc |
| PyxzeKpnI | gatcggtaccttcgcgtcactcccttttttcac |
| PfosbBam | gatcggatccacaggaaaggaagggtttatgg |
| PfosbKpnI | gatcggtaccaaatcattccccccttgaaagc |
| PhrcaEcoR | gatcgaattctcgacctgattacgacagcgcc |
| PhrcaKpnI | gatcggtacccatcatcacctctgttagcactc |
| PybfqBam | gatcggatccaaagcagagttgctctcccaat |
| PybfqKpnI | gatcggtaccgttttaaacaccctgttcaattatag |
