# Supplementary material for: Promoter Screening from Bacillus subtilis in Various Conditions Hunting for Synthetic Biology and Industrial Applications
Source: PLoS One. 2016 Jul 5;11(7):e0158447. doi: 10.1371/journal.pone.0158447 (PMC4933340; doi:10.1371/journal.pone.0158447)
Supplement: S2 Table — (DOCX) [file pone.0158447.s003.docx]

**S2 Table Predicted -10 boxes, -35 boxes, spacers and regulated sigma factors of these promoter candidates.**

| Promoter | Predicted -35 box and -10 box | spacer | Sigma factor |
| --- | --- | --- | --- |
| PmreBH | cccccaaaaatcgcagtatttctgagaaactttaaatgtagaatcatattaaattagg | 14 | sigI |
| PywaC | taataaaaaagaccgctcgtttcatgcggtctttttttgttacaatcgaccgcattttgta | 14 | sigMW |
| PytpA | ggtggatatctggcttgtgttccttataagggtagatcataagattcatccttctttctct | 14 | sigM |
| PybfO | gcttccggttgagacatttttacaaaatacgtatcggttttactttcatcaggtgatgag | 14 | sigW |
| PyuaF | atcatttataaagcacttcaaatgggttatttgtcttaaaattttgaaacttttccc | 14 | sigW |
| PylxX | tcgctggaggcctaattgatcagctgtcaatggatcatatttctgtgtcggcaga | 14 | sigE |
| Pspo0M | ctgcaaccaaactttctttattcacttccatcgactcctttattttcaatattattcttagt | 14 | sigHW |
| PmreB | ccaaaaatcgcagtatttctgagaaactttaaatgtagaatcatattaaattaggata | 14 | sigA |
| PyceC | cgaggtttttcgtcaattattcttaacttttacgaaactttgatataataacaaacgtatat | 15 | sigBMWX |
| PyacL | cagacaactgtgatttcggttaaaaccttatgaatacgggtatattaatgttggtttttg | 15 | sigBM |
| PhrcA | aattattaggcaatgaagtttttggcgcttttttgggtgagttataattgacatttttcttg | 15 | sigA |
| PybgB | atgtcggagcgcctgcgtttttatcagaatcccttacctatgataaaaatgaagaagt | 15 | sigAY |
| PyxjI | tatttcaaacgaaaaggctgctgaatcacgcagccttttaaaattgttttgatttagag | 15 | sigBW |
| PfosB | cataatggtcatgttactattttaaacagtccgtttttgtttacactggtatttttttcagctg | 15 | sigW |
| PpssA | aagattggcattcatttttcaaaaggtaacaaatgttgtataataatagaatttgaatgc | 15 | sigAX |
| PclpE | ctgctttgagcatattggtttgaatgccgttaagtttgccgtatactaatagtcaaagaa | 16 | sigA |
| PsigV | ttttttatgcataaaccccccatgctgatttacttctctcttttaaaaaatgtgattctgcgc | 16 | sigV |
| PhtrB | aacgtcattataaactagttttaacatacggcaggcaattttcataatttcacatattcttt | 16 | unknown |
| PsigX | tgtttttttaatagccaacattaataaaatttaaggatatgttaatataaattcccttccaa | 16 | sigAX |
| PyeaA | gagcatgaagactatgacatgatgagaccgatggatataactgatccccataaataa | 16 | sigWE |
| PdivIB | atcgctgattcaagttctgactgaagctgttcatatgatatactgtaagcaaacgaca | 16 | sigEMA |
| PyceE | cgtttgtgagctttacagacacggcggcgagtggaaattcaatgcaatcggcagcg | 16 | sigBMWX |
| PsigX | aatagccaacattaataaaatttaaggatatgttaatataaattccctt | 16 | sigAX |
| PradA | tatagaagacggaaatgaggcatacagcatgtaagtgtatgcctcactttcatatca | 17 | sigAB |
| PgroES | atcttatcacttgaaattggaagggagattctttattataagaattgtg | 17 | sigA |
| PtrnQ | gaaatagagggttgttatttgaaaggaattatcgtataattagttgtgct | 17 | unknown |
| PdltA | tatgtatggttttcacaccgcgaataccggttcatatatttataacgatt | 17 | sigDMXV |
| Pclpp | aggccagctttttgtttgacctttattgaccaaaaatgtatcatgtaactacatactt | 17 | sigB |
| PsigI | tcaatatttctttttctcctttgcgaatccctatcaaattagctatcattaatgagtagttat | 17 | sigI |
| PhtpG | atgagtgagaaaaagagtgaaaacaaatccaactaatgttaaatttacatatatatgca | 17 | sigA |
| PyqeZ | caaagaaaaggacatctttctaagagagatgtctttttttatacataaaaaaatgaaac | 17 | sigW |
| PmurB | gacgcggctgcccgtttatacagcgtactcgaggaattaaaaaaatagtagaaaagc | 17 | sigM |
| PfabHA | gtttagacgaatatattgccatgtgaaaaaaaataggatagaattagtacctgatacta | 17 | sigA |
| PaprE | tctcaaaaaaatgggtctactaaaatattattccatctattacaataaattcacagaatag | 17 | sigA |
| PywnJ | tgaagaaaacgtctatccttgtgatgggcgtttttttctgttactttttgctttgttttaccg | 17 | sigFMWX |
| PypuD | cagaaatgtgaaacatattcccgttatgcatcgttatattaataatttacgagaatttacg | 17 | sigBM |
| PbcrC | agacaatctctatttttatttgaaacttttcatgagtaagattagtctactaaatataaaaat | 17 | sigMIWX |
| PmetA | aacacgtatagatagattgaaaattggcgtgaaaattcctataatgaagaaaattaaac | 17 | sigM |
| PcsbB | gttttttttcatagtctgttttaaacatgacaaaataacattaaaaatcatgaatgtcaccat | 17 | sigBX |
| PybfQ | cagtctgggcggatctgttcatctttgcggcattttcctgtatgatatagtaagagtgtgc | 17 | sigW |
| PydjO | gaagagccagatgcttttaacagcacactcctctcctttttagatggggttattggaaagt | 17 | sigW |
| PyknW | aggtttttggaaatttattagaaaaacatgaaactttttgatatccttcccgtactatttgtta | 17 | sigW |
| PydbS | tcagcaaactttcagctgacggaagccatttttctctgtatgcttaagaatgaaaccttt | 17 | sigW |
| Pabh | aaagcgggaaactttttcaaagtttcattcgtctacgatatattgaggtacatccttaaa | 17 | sigXM |
| PysdB | tgtttttacagctttcttttagaaaaagtgaaacctttttctatgcttttcgtattacatcaga | 18 | sigBW |
| PftsH | cttccgagatggttattgtttgtattggaatgattttctatggtactattgaacatagttgtg | 18 | sigAM |
| PywbO | ggcttctttattaaaaaacagttgtaactttaattgttacagtttaaaatcaatttagtatga | 18 | sigXW |
| PdnaJ | ccgggataaaaggaattgaaaaatcataattcaaaatgatacaatctaatttatgtgag | 18 | sigA |
| PyjbC | tatagatgtaaggctgtttaaacaagaagaaatggggtatatctaaaagtatgaagac | 18 | sigWBXM |
| PythP | cagccatccttgttggattactatgtatttcggctcaatattattatggttaaagaaacttt | 18 | sigW |
| PminC | agtctaaagaaagaattgagagatgagtaaaaaggattttatctttttttgacgaaatga | 18 | sigH |
| PdltE | ttcatcctgagcatctcataggacgcggctgcatatttcctgctaagatttgtaatagta | 18 | sigDMXV |
| PclpC | aacgaacttatgattttgacgcagccagggtttttacagcaatactcgggaggcgcttt | 19 | sigBF |
| PugtP | ttttctttttttagtgatacgctgagcaaaaaatatgataaaatacatctgatt | 19 | sigM |
| PyrhK | aaggggcaaaaactttgactgctcttattgttcaggcgatgaaatcgtaaaaaagagat | 19 | sigV |
| PycbR | aagtcacactgcttttatcagccgacggcggagacgtgtgaaaatgtatataacacatt | 19 | unknown |
| PsigW | tttttcttttcggaaaaatcattccaacttctaactgttcagtctgtataataattttaaaaatat | 19 | sigW |
| PmurF | gattaagcatacattctaatgaagaaggacactgccggtaaaagggcagtgttttttccc | 19 | sigM |
| PugtP | ctaatttttctttttttagtgatacgctgagcaaaaaatatgataaaatacatctgattcagt | 19 | sigM |
| PyxzE | gaatcgatcactttgaaatgaaaccggtcagcgtttcatccgtataacagatatggtgaa | 19 | sigW |
| PyvlA | ctaaagaatgaatttgaaacctgaagagattttaaacgtataaataagtaaaacgtccct | 19 | sigW |
| ProdA | tttttcatttgaaaagttttgtgtcaatcgaaacatttcggtttatgatacgtcatatttcgtgt | 19 | sigM |
| PsppA | agctttctcatgaagtgaaacatttttcatattgaatcgtataatgagagagtttaggaaa | 19 | sigW |
| PyusI | ttaagaagcactgttttaggtttttaaaacaaactgtgttaaaataacctcgattattt | 20 | sigA |
| PcssR | tgtgaaattatgaaaattgcctgccgtatgttaaaactagtttataatgacgttgaaagga | 20 | sigA |
| PywrK | caatctcttttttcttttgggattcttatggaatttttattaatttaatataatcgcttagtacaac | 20 | unknown |
| PyjoB | gggataaaaaatggctgacaatttgattggattggcacattatgatctcctccatatgtaa | 20 | sigW |
| PyoaG | tttggccaatcaggagaggcgttttcagccagcgtattatctaataaaagaacgtc | 20 | sigW |
| PoatA | cgtcatttcaaacttgctcgtgggagaacgttatattcgataaaataaaaacatccatctaa | 21 | sigV |
| PydaH | aacaatgctttatgaatctaacccgcctgacttggcggatgatataatctttatttgcgtata | 21 | sigM |
| PsecDF | cagataagaagtggaaaaagaaaacggaactgagcatttatgaactggcgagtgatat | 21 | sigM |
| PypbG | aaataaaatggggcgttttctcaaacgatagaaacgtctttttttattgtccgtcttattgat | 21 | sigM |
| PtilS | ctttgcttggaggggttgaaagtgttaggatatgaatatctttaaaatacctgctcattgtga | 21 | sigM |
| PpspA | gggcatctttttttattgcttacatttttgttcaggtaaaaagttgtatattttttgtttgaaatgtc | 21 | sigW |
| PdivIC | tgtttgaaacttcttcctgtgaaaatgcgtctaacttttagacgttattttgaaaaaggtgcag | 21 | sigEMXW |
| PypuA | ttactaaaaaataaaaatgaaacctgaatctctttatctccgtctaacctaacggaactgtat | 22 | sigM |
| PyozO | gaaatataaaacaaaattaaaaaaatcattaaaaaccacttttttataaaaacattgctgaat | 22 | sigW |
| PhtrA | tttcacaatttcccataatcttttcatttttatcccacagtttttgtttatgataaactcaagtcat | 22 | sigA |
| PyrhH | gtattcccttatcattggcattagaaagagggcagaaaacattatgttttctgcttttttttcg | 22 | sigWMX |
| PybfP | aaacaggggatttttacatatcgcaaaaataatcaaaacatataatccgtgatccattattc | 22 | sigW |
| PyceG | ccgcactctgtattcttcattatcattatcgctgcatttgcggtaacattgatcattcactaca | 22 | sigBMW |
| PxpaC | tacaccgccattatattcatagacctgaaaaggtctttttttgtactcttaataataaaaagaa | 22 | sigWK |
| PyoaF | ggtttgaaaaataatgaaacccggagtatgccaagcccgtataacataacatcgacatag | 22 | sigW |
| Pddl | tttgttgtgatttttttcatatgtcacatctccttttcaatgccattttaatataaaataaaaagga | 22 | sigM |
| PyngC | tgcggtaaaaaaattgtaaccatcttgctcgtcaatcagtcatataatagtgaaaaagatta | 23 | sigM |

Predicted -10 and -35 boxes were obtained from:

<http://linux1.softberry.com/berry.phtml?topic=bprom&group=programs&subgroup=gfindb>

σ-dependent information of promoter candidates was from:

<http://www.subtiwiki.uni-goettingen.de/wiki/index.php/Categories>
