# Supplementary material for: Promoter Screening from Bacillus subtilis in Various Conditions Hunting for Synthetic Biology and Industrial Applications
Source: PLoS One. 2016 Jul 5;11(7):e0158447. doi: 10.1371/journal.pone.0158447 (PMC4933340; doi:10.1371/journal.pone.0158447)
Supplement: S3 Table — (DOCX) [file pone.0158447.s004.docx]

**S3 Table Functions of proteins encodes by genes downstream of these promoter candidates and their categories**

| Gene | Annotation |
| --- | --- |
| Heat shock proteins | |
| *ftsH* | ATP-dependent zinc metalloprotease FtsH |
| *radA* | K04485 DNA repair protein RadA/Sms |
| *clpP* | K01358 ATP-dependent Clp protease, protease subunit [EC:3.4.21.92] |
| *yacl* | yacL; PIN and TRAM-domain containing protein YacL |
| *dnaJ* | K03686 molecular chaperone DnaJ |
| *clpC* | K03696 ATP-dependent Clp protease ATP-binding subunit ClpC |
| *cssR* | K07770 two-component system, OmpR family, response regulator CssR |
| *hrcA* | K03705 heat-inducible transcriptional repressor |
| *sigI* | K03093 RNA polymerase sigma factor |
| *clpE* | K03697 ATP-dependent Clp protease ATP-binding subunit ClpE |
| *groES* | K04078 chaperonin GroES |
| *htpG* | K04079 molecular chaperone HtpG |
| *htrB* | K04771 serine protease Do [EC:3.4.21.107] |
| *mreBH* | K03569 rod shape-determining protein MreB and related proteins |
| *htrA* | serine protease Do-like HtrA |
| *bcrC* | K19302 undecaprenyl-diphosphatase [EC:3.6.1.27] |
| Cell envelope stress proteins (controlled by SigM, V, W, X, Y) | |
| *abh* | transition state regulator Abh |
| *csbB* | glycosyltransferase CsbB (EC:2.4.-.-) |
| *divIB* | K03589 cell division protein FtsQ |
| *dltA* | K03367 D-alanine--poly(phosphoribitol) ligase subunit 1 [EC:6.1.1.13] |
| *fabHA* | K00648 3-oxoacyl-[acyl-carrier-protein] synthase III [EC:2.3.1.180] |
| *fosB* | K11210 metallothiol transferase [EC:2.5.1.-] |
| *minC* | K03610 septum site-determining protein MinC |
| *mreB* | rod shape-determining protein MreB |
| *pbpE* | penicillin-binding protein 4* |
| *pssA* | CDP-diacylglycerol--serine O-phosphatidyltransferase (EC:2.7.8.8); |
| *pspA* | K03969 phage shock protein A |
| *rodA* | rod shape-determining protein RodA |
| *sigV* | K03088 RNA polymerase sigma-70 factor, ECF subfamily |
| *sigW* | K03088 RNA polymerase sigma-70 factor, ECF subfamily |
| *sigX* | K03088 RNA polymerase sigma-70 factor, ECF subfamily |
| *ylxX* | hypothetical protein |
| *sppA* | K04773 protease IV [EC:3.4.21.-] |
| *xpaC* | 5-bromo 4-chloroindolyl phosphate hydrolysis protein XpaC |
| *ybfP* | AraC family transcriptional regulator |
| *ybfO* | hydrolase YbfO |
| *YbfQ* | K07146 UPF0176 protein |
| *ybgB* | membrane protein |
| *yceC* | stress response protein SCP2 |
| *yceE* | hypothetical protein; K05795 tellurium resistance protein TerD |
| *yceG* | hypothetical protein |
| *ydbS* | K09167 hypothetical protein |
| *yeaA* | hypothetical protein |
| *ydjO* | hypothetical protein |
| *yjbC* | acetyltransferase YjbC |
| *yknW* | hypothetical protein |
| *yoaG* | hypothetical protein |
| *yozO* | hypothetical protein |
| *ypuA* | hypothetical protein |
| *yqeZ* | hypothetical protein |
| *yrhH* | methyltransferase |
| *yrhK* | hypothetical protein |
| *ythP* | K01990 ABC-2 type transport system ATP-binding protein |
| *ytpA* | K01048 lysophospholipase [EC:3.1.1.5] |
| *yuaF* | membrane protein |
| *yvlA* | hypothetical protein |
| *ywbO* | hypothetical protein |
| *ddl* | K01921 D-alanine-D-alanine ligase [EC:6.3.2.4] |
| *murF* | K01929 UDP-N-acetylmuramoyl-tripeptide--D-alanyl-D-alanine ligase [EC:6.3.2.10] |
| *divIC* | K13052 cell division protein DivIC |
| *metA* | K00651 homoserine O-succinyltransferase [EC:2.3.1.46] |
| *murB* | K00075 UDP-N-acetylmuramate dehydrogenase [EC:1.3.1.98] |
| *oatA* | peptidoglycan O-acetyltransferase YrhL (EC:2.3.1.-) |
| *secDF* | K12257 SecD/SecF fusion protein |
| *spo0M* | K06377 sporulation-control protein |
| *tilS* | K04075 tRNA(Ile)-lysidine synthase [EC:6.3.4.19] |
| *ugtP* | K03429 processive 1,2-diacylglycerol beta-glucosyltransferase [EC:2.4.1.157 2.4.1.315] |
| *ydaH* | hypothetical protein |
| *yjoB* | ATPase YjoB |
| *yngC* | membrane protein |
| *yoaF* | hypothetical protein |
| *ypbG* | hypothetical protein |
| *ypuD* | hypothetical protein |
| *ysdB* | sigma-W pathway protein YsdB |
| *ywaC* | K07816 putative GTP pyrophosphokinase [EC:2.7.6.5] |
| *ywnJ* | membrane protein |
| *yxjI* | hypothetical protein |
| *yxzE* | hypothetical protein |
| Resistance against toxic metals (based on similarity) | |
| *ywrK* | K03893 arsenical pump membrane protein |
| *yusI* | hypothetical protein |
| *ycbR* | hypothetical protein |
| Other kind of genes | |
| *trnQ* | trnQ-Arg; tRNA-Arg; K14219 tRNA Arg |

Function of genes downstream of promoter candidates were obtained from:

<http://www.kegg.jp/kegg-bin/show_organism>
